# 2200: Best practices for social and behavioral research: A new course to address good clinical practice and preliminary course evaluation

**DOI:** 10.1017/cts.2017.165

**Published:** 2018-05-10

**Authors:** Susan Lynn Murphy, Christy Byks-Jazayeri, Brenda Eakin, Jordan Hahn, Brandon Lynn, Elias M. Samuels, Fanny Ennever, Sarah Peyre, Margarita L. Dubocovich, Wajeeh Bajwa

**Affiliations:** University of Michigan School of Medicine, Ann Arbor, MI, USA

## Abstract

OBJECTIVES/SPECIFIC AIMS: To conduct a preliminary evaluation of the Social and Behavioral Research Best Practices Course. METHODS/STUDY POPULATION: Learners are sampled from 5 institutions: University of Michigan, University of Rochester, University of Florida, Boston University, and University of Buffalo. Learners who take the course and consent to be in the study receive a web link to a survey immediately after course completion and at 2–3 months follow up. In addition to demographic information, learners will report their perceptions of usefulness and relevance of the course to their job, their satisfaction with the course and associated job aids, and at follow-up, if and how the course impacted their work. Additional information will be collected from the learning management systems which host the course at each institution. The data collected will include the number of participants who take the course, the number who complete, how many times the course was attempted, and pass rates. RESULTS/ANTICIPATED RESULTS: We anticipate that several hundred learners will take the course by the end of our project. Of learners who agree to participate in the survey, we anticipate that they will find the course useful and relevant to social and behavioral clinical trials and will be satisfied with the course. Information including suggestions about missing content, items or content that were not extremely clear, or any other comments will be collected to iterate and expand the course. DISCUSSION/SIGNIFICANCE OF IMPACT: This course was developed to fill a gap in training in good clinical practice for social and behavioral research. An evaluation of how the training provided in the course impacts the jobs of learners is needed both to ensure that the most relevant information is included in the course as well as to identify ways that the training may contribute to the quality and safety of social and behavioral clinical trials.

